# Treatment of Liver Fibrosis: A 20-Year Bibliometric and Knowledge-Map Analysis

**DOI:** 10.3389/fphar.2022.942841

**Published:** 2022-07-12

**Authors:** Yun-Kai Dai, Zhi-Min Zhao, Chenghai Liu

**Affiliations:** ^1^ Institute of Liver Diseases, Shuguang Hospital Affiliated to Shanghai University of Traditional Chinese Medicine, Shanghai, China; ^2^ Shanghai Key Laboratory of Traditional Chinese Clinical Medicine, Shanghai, China; ^3^ Key Laboratory of Liver and Kidney Diseases, Ministry of Education, Shanghai, China

**Keywords:** treatment of liver fibrosis, Citespace, VOSviewer, bibliometric, knowledge-map

## Abstract

**Objectives:** To analyze the research hotspots, evolution, and trends of the treatment of liver fibrosis in the recent 20 years, bibliometric and knowledge-map analysis were used.

**Methods:** Publications associated with the treatment of liver fibrosis were retrieved from the Web of Science Core Collection on 16 April 2022. CiteSpace 5.8.R3 and VOSviewer 1.6.18 were calculated to perform bibliometric and knowledge-map analysis.

**Results:** A total of 72,686 authors from 200 institutions in 134 countries/regions published 15,237 studies in different academic journals. United States was the most productive country, and Shanghai Jiao Tong University was the most published institution. Trauner Michael had the most published articles, whereas Scott L. Friedman was the most frequently co-cited author. Moreover, there was frequent inter-institution cooperation between countries in the years 2015 and after, but the before years showed rare inter-institution cooperation. The journal *HEPATOLOGY* was both the most published publication and the most frequently co-cited one in this field. Screened keywords, such as virus infection, inflammation, oxidative stress, activation of hepatic stellate cell (HSC), and hepatocellular apoptosis, could be both therapeutic targets and pathological mechanisms in terms of liver fibrosis. Furthermore, long-term suppression of hepatitis B virus replication and the activation of HSC were the latest hotspots and topics related to the treatment of liver fibrosis. Besides, the treatments of nonalcoholic fatty liver disease and nonalcoholic steatohepatitis were also involved in the treatment of liver fibrosis, which were both emerging topics and rapidly developing hot fields.

**Conclusion:** This bibliometric analysis conducted a full overview of the treatment of liver fibrosis, which provided important clues and ideas for scholars focusing on this field. Not only that, the field is still in a stage of rapid development and will continue to be a research hotspot in the future.

## Introduction

Liver fibrosis, with the characteristics of the recruitment of activated inflammatory cells and progressive deposition of extracellular matrix (ECM) proteins, is a common endpoint of chronic hepatocellular damage (Lambrecht et al., 2020). If this fibrotic disease is not controlled in time, it will worsen distortion of the physiological architecture of the liver, resulting in the onset of bridging fibrosis and cirrhosis (Roehlen et al., 2020). Because of asymptomatic characteristics, the progression of liver fibrosis to cirrhosis is often ignored, over time easily leading to the eventual deterioration of the liver function and enhanced risk of hepatocellular carcinoma (HCC). To our knowledge, liver fibrosis and cirrhosis are the initiating factors of mortality for the majority of chronic liver diseases worldwide (Schuppan et al., 2018; Zhao et al., 2021). Therefore, their prevention and reversal are crucial solutions for clinicians.

Since then, efforts involving in the treatment of liver fibrosis have gotten remarkable progress. Currently, targeted antifibrotic strategies, such as antiviral therapies and lifestyle changes in alcoholic or nonalcoholic fatty liver disease (AFLD or NAFLD) (Lambrecht et al., 2020; Li, 2020), include etiological treatments and symptomatic treatments. Besides, because of unique advantages in treating liver fibrosis, traditional Chinese medicine (TCM) has also been tremendous progress in anti-liver fibrosis (Liu et al., 2003; Liu et al., 2005; An et al., 2014; Qu et al., 2014; Li et al., 2021). However, no article mentioned above comprehensively reported the research hotspots and evolving trends related to the treatment of liver fibrosis.

Bibliometrics, an approach to systemically reviewing a research field, can analyze literature quantitatively using mathematical and statistical methods. Through bibliometric analysis of publications on a specific topic, influential and effective areas, knowledge base, and emerging topics in scientific research can be identified (Liu et al., 2005; Chen and Song, 2019; Ke et al., 2020). It seems that the advantages of this analysis are unmatched by other methods such as review, meta-analysis, or experiment research. Considering its strengths and in order to outline the knowledge domain and emerging trends in the treatment of liver fibrosis, this study would use CiteSpace [version 5.8.R3 (64-bit)] and VOSviewer (version 1.6.18) tools to draw the scientific knowledge-maps and analyze publications from 2002 to 2021.

## Materials and Methods

### Data Selection

Relevant data were retrieved on 16 April 2022 from the most influential database [Web of Science Core Collection (WoSCC)]. The retrieval time span was from 1 January 2002 to 31 December 2021. The limitation of language was English and the restriction of article type was Article or Review. Based on the Boolean logic operator, search terms included “treatment of liver fibrosis” OR “liver fibrosis treatment” OR “treatment of hepatic fibrosis” OR “hepatic fibrosis treatment.” Results of retrieval were selected in the form of “Full Record and Cited References” and downloaded in the document format of “Plain Text.” Then, given that this file format of “download_*.txt” was only recognized by CiteSpace, the downloaded files were renamed after it. Meanwhile, in this study, there was no need to apply for ethical approval because data were directly retrieved and exported from the WoSCC database.

### Data Analysis and Visualization

Nowadays, bibliometric software used widely consists of CiteSpace, VOSviewer, UCINET, SciMAT, Pajek, and Bicomb (Qin et al., 2020). However, no consensus has yet been reached on which software is the best one. Considering their individual characteristics and combing them with our actual needs, CiteSpace [version 5.8.R3 (64-bit)] and VOSviewer (version 1.6.18) (van Eck and Waltman, 2010; Xu and Feng, 2021) were used for bibliometric analysis in this study.

CiteSpace, a citation visualization software based on Java and developed by Prof. Chaomei Chen, is a bibliometric analysis tool that can analyze the potential research hotspots and trends using knowledge-map in a certain field (Chen, 2004). In this study, the annual growth trend of publication outputs, countries/regions and institutions, journals and co-cited journals, authors and co-cited authors, occurrence of keywords, co-cited references, and reference burst were analyzed and visualized using CiteSpace [version 5.8.R3 (64-bit)].

VOSviewer, a bibliometric mapping software based on Java and developed by Leiden university, is good at the visualization of scientific knowledge and handling large bibliometric maps based on network data (van Eck and Waltman, 2010). According to the bibliographic and text data, productive journals and authors, relevant knowledge-maps, keyword researches, and cluster maps were analyzed and visualized as a supplement to the deficiency of CiteSpace.

Besides, Microsoft Office Excel 2010 was used for the management of the WoSCC database, analysis, and visualization of the annual publications.

## Results

**FIGURE 1:**
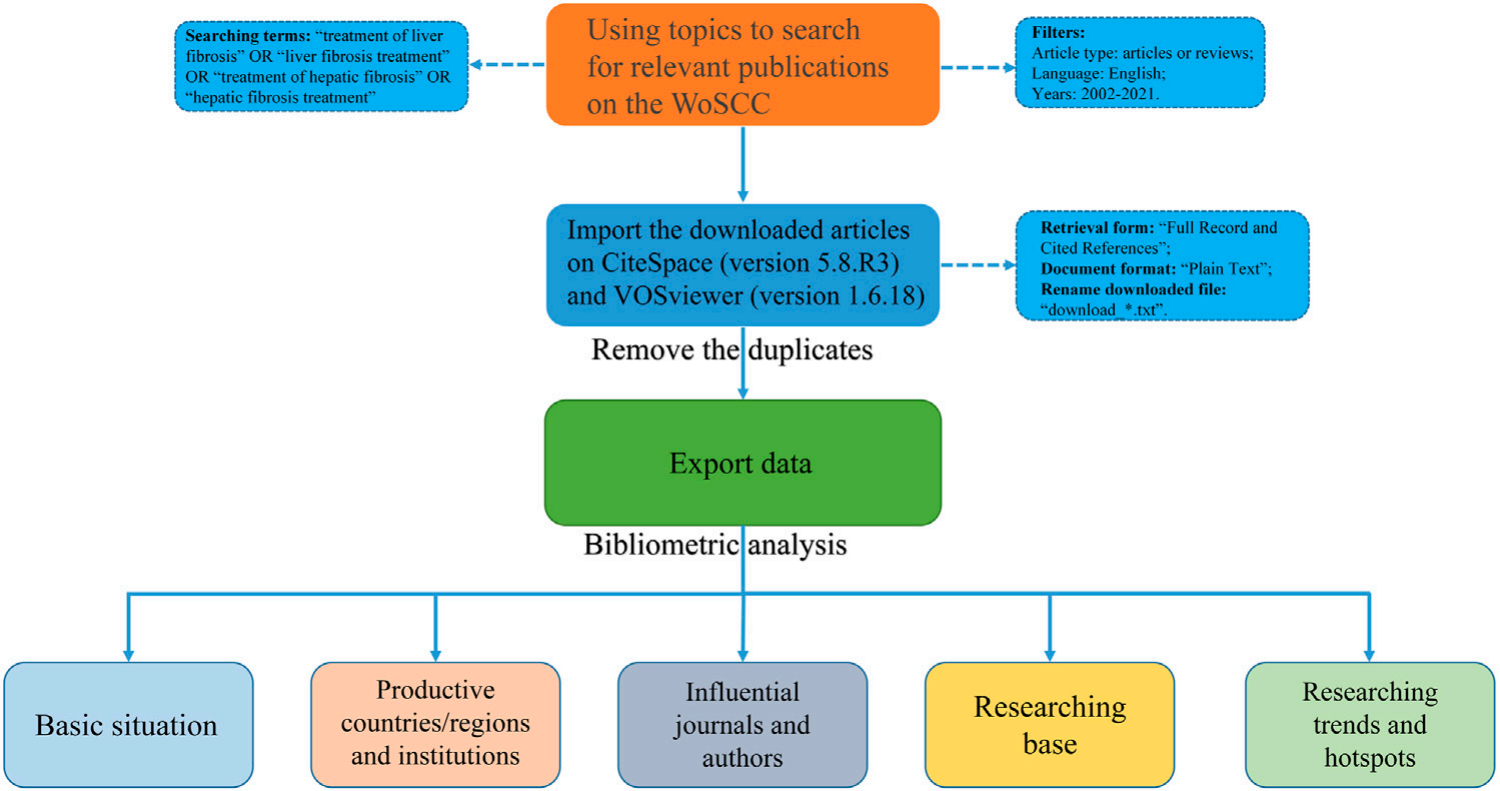
Flowchart of this study.jpg.

The flow chart of bibliographic retrieval and research steps in this study are illustrated in Figure 1.

### Annual Growth Trend of Publications

**FIGURE 2:**
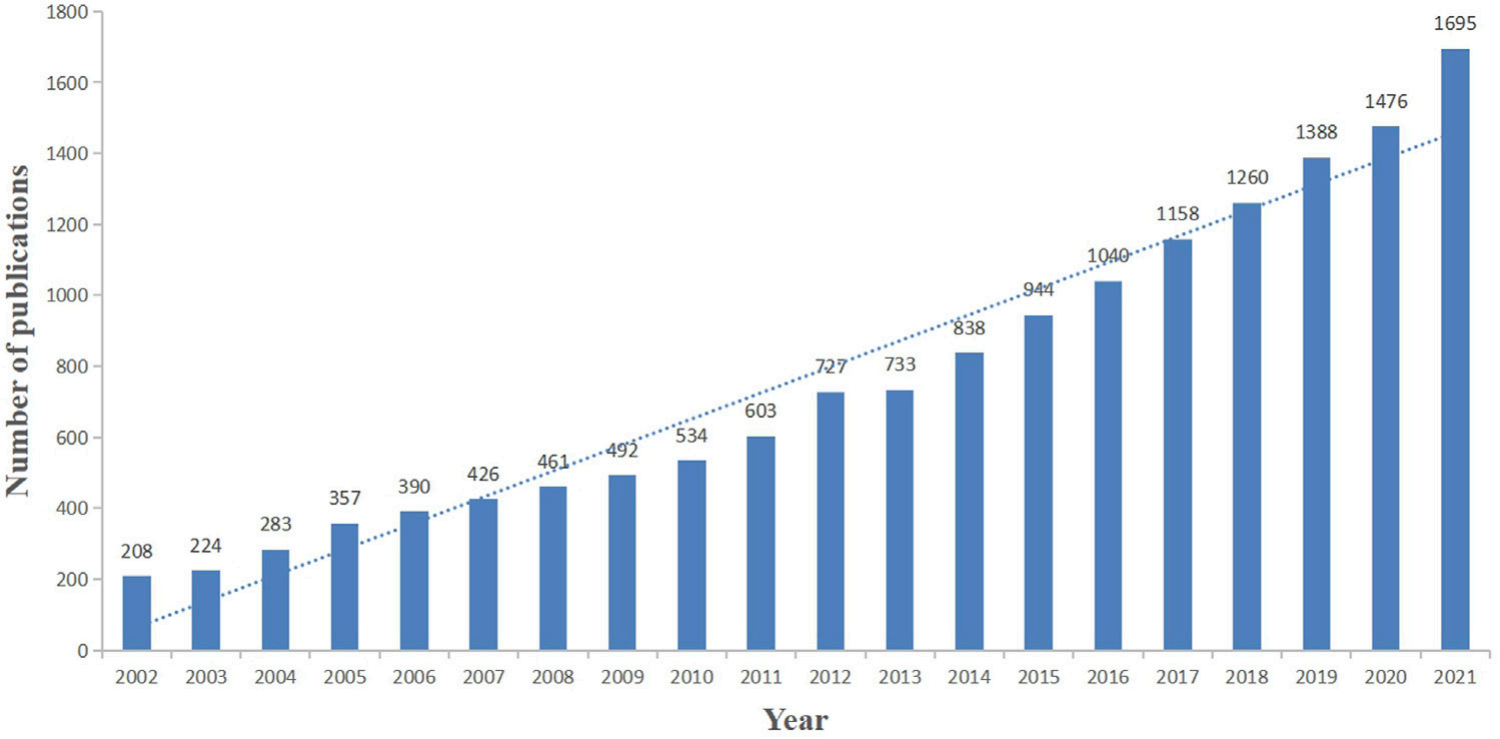
Chronological trend of publications about the treatment of liver fibrosis.jpg.

Based on data selection criteria, a total of 15,237 studies involving in the treatment of liver fibrosis were retrieved from 2002 to 2021 on WoSCC (Annexes 1), which included 12,717 (83.5%) original articles and 2520 (16.5%) reviews. As shown in Figure 2, the number of publications about the treatment of liver fibrosis rises steadily and gradually year by year.

### Productive Countries/Regions and Institutions

A total of 200 institutions from 134 countries/regions co-authored 15,237 studies. As shown in Table 1, as for the number of output publications, United States (*n* = 3687, accounting for 24.61% of the total) ranked first, followed by China (*n* = 3254, 21.72%), Japan (*n* = 1057, 7.05%), Italy (*n* = 907, 6.05%), and Germany (*n* = 882, 5.79%). Meanwhile, we concluded from Table 1 that the number of publications from the United States and China was far beyond other countries/regions. Among the top 10 nations, four-fifths of them were developed countries. And United States had higher centrality with 0.84, indicating that the country played an important role as a bridge in the cooperation between countries. As for the institutions, Shanghai Jiao Tong University (*n* = 201, 1.34%) with the most publications ranked first, followed by University California San Diego (*n* = 191, 1.28%), Mayo Clinic (*n* = 190, 1.27%), Fudan University (*n* = 154, 1.03%), and Cairo University (*n* = 143, 0.95%). However, four-fifths of the top 10 institutions, it should be noted, were from China and United States.

**TABLE 1:** The top 10 countries/regions and institutions involved in the treatment of liver fibrosis.

Rank	Country/region	N (%)	Centrality	Year	Institution (country/region)	N (%)	Centrality	Year
1	United States	3687 (24.61%)	0.84	2002	Shanghai Jiao Tong Univ (China)	201 (1.34%)	0.05	2007
2	China	3254 (21.72%)	0	2004	Univ Calif San Diego (United States)	191 (1.28%)	0.05	2005
3	Japan	1057 (7.05%)	0	2007	Mayo Clin (United States)	190 (1.27%)	0.07	2003
4	Italy	907 (6.05%)	0.06	2005	Fudan Univ (China)	154 (1.03%)	0.01	2004
5	Germany	882 (5.89%)	0.05	2004	Cairo Univ (Egypt)	143 (0.95%)	0.01	2011
6	England	675 (4.51%)	0.04	2007	Harvard Univ (United States)	138 (0.92%)	0.09	2002
7	France	670 (4.47%)	0.08	2007	INSERM (France)	128 (0.85%)	0.03	2002
8	South Korea	641 (4.28%)	0	2005	Sun Yat Sen Univ (China)	126 (0.84%)	0.01	2004
9	Spain	616 (4.11%)	0.02	2007	Capital Med Univ (China)	123 (0.82%)	0.02	2009
10	Egypt	478 (3.19%)	0.04	2004	Johns Hopkins Univ (United States)	122 (0.81%)	0.08	2003

**FIGURE 3:**
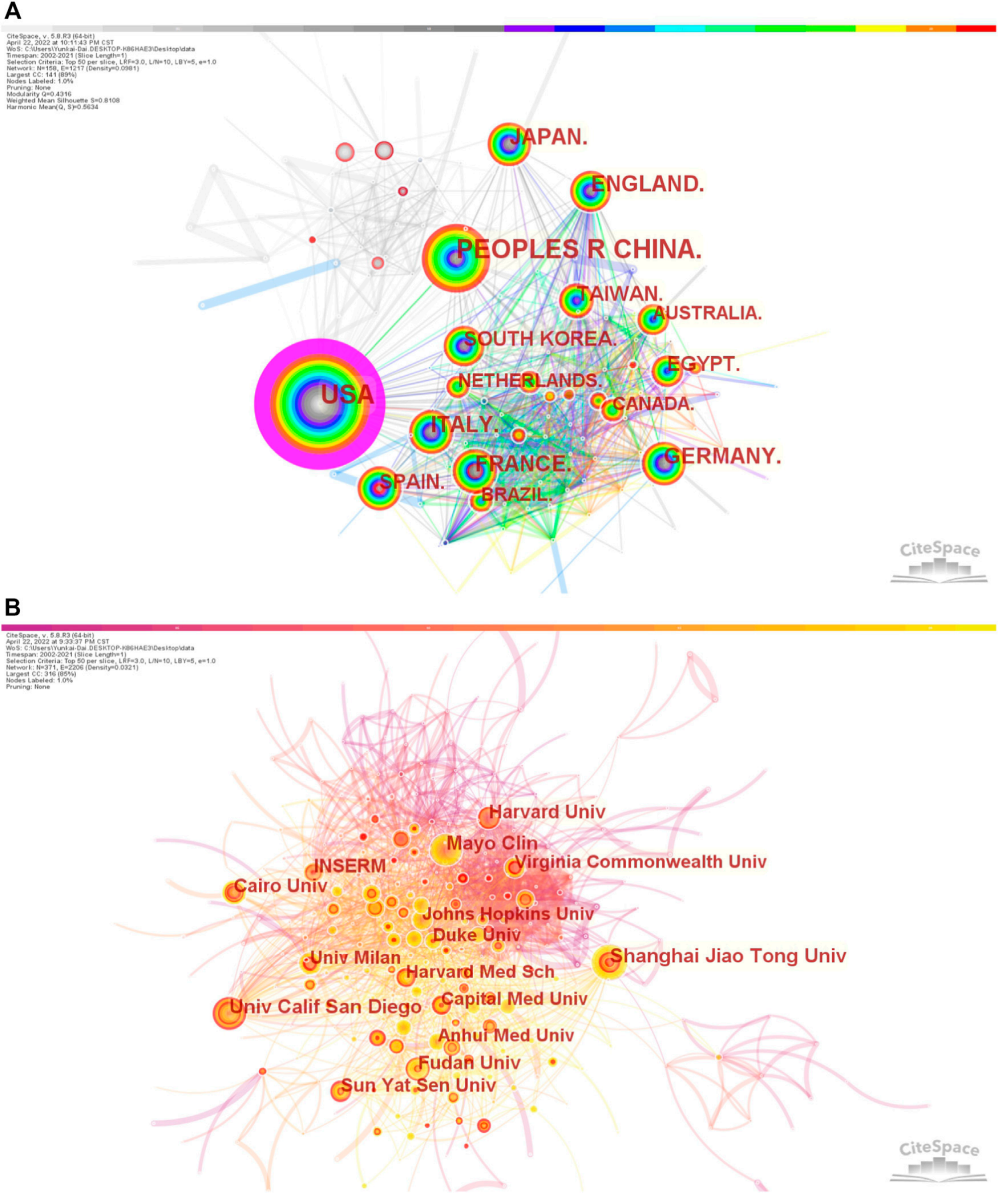
Co-occurrence maps **(A)** Countries; **(B)** Institutions. Notes: The size of the node represents the co-occurrence frequencies while the links reflect the co-occurrence relationships. The color of node and line indicates different years.jpg.

As displayed in Figure 3A (co-occurrence map of countries), the connections between countries were strong, suggesting that there were a lot of cooperations, especially United States and China. As shown in Figure 3B (co-occurrence map of institutions), the connections of years colored by orange and yellow were the most widely distributed, indicating that 2015 and after were the most intensive years of inter-institution cooperation, but there was rare inter-institution cooperation before 2015.

### Productive Journals and Co-Cited Journals

**FIGURE 4:**
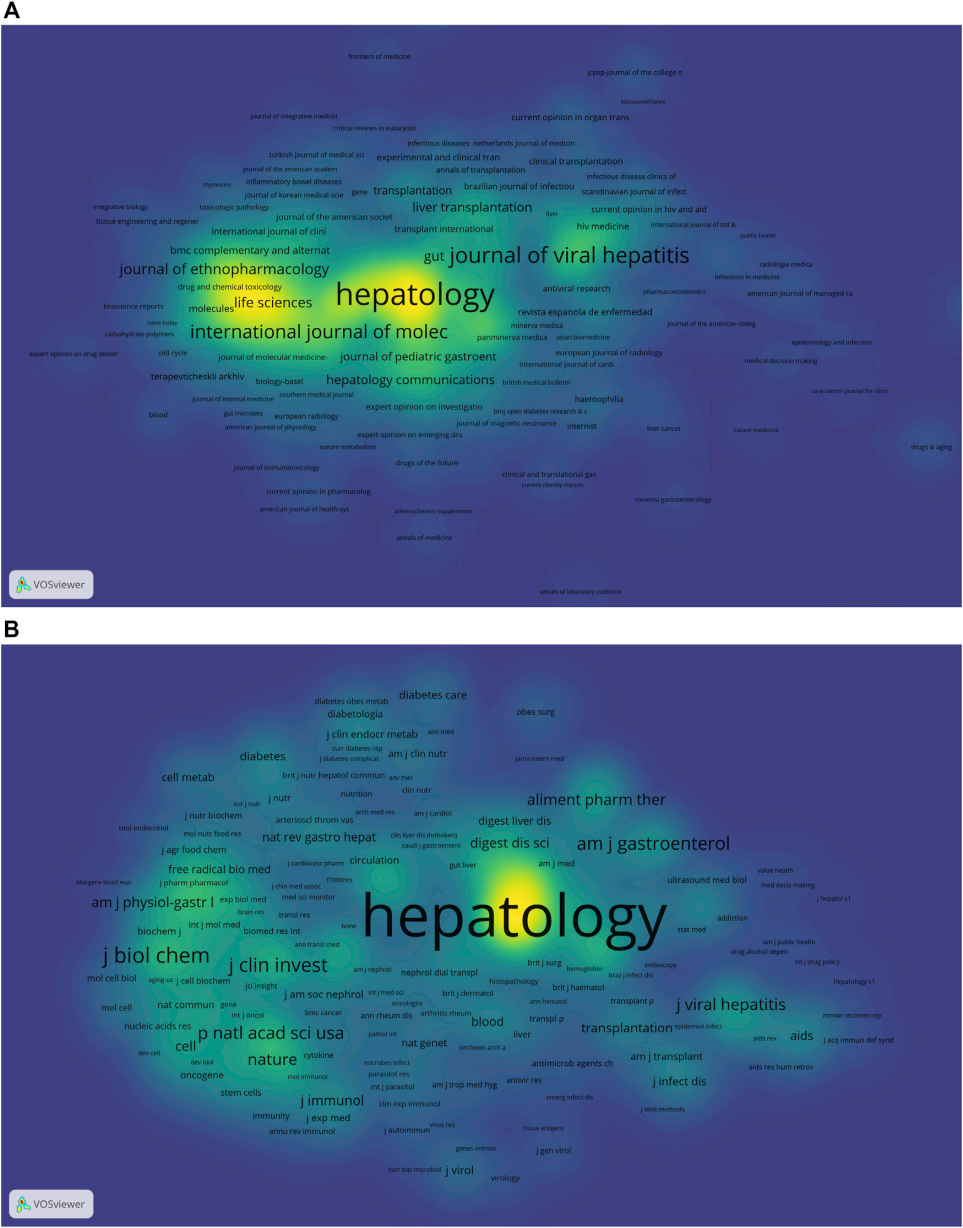
The density maps **(A)** Journals; **(B)** Co-cited journals. Notes: The size of the word and round, and the opacity of yellow are positively associated with the co-cited frequency.jpg.

In order to observe the journals with the most number of published publications and that of co-citations in the treatment of liver fibrosis, VOSviewer (version 1.6.18) and CiteSpace [version 5.8.R3 (64-bit)] were used for the co-citation and co-cited journal analyses. Statistical results suggested there were 15,260 documents published in different academic journals (*n* = 2017). Table 2 showed the top 10 journals and co-cited journals associated with the treatment of liver fibrosis. Among these journals, *HEPATOLOGY* with 454 citing articles was the most published publication, followed by *WORLD J GASTROENTEROL* (*n* = 419), *J HEPATOL* (*n* = 393), *PLOS ONE* (*n* = 381), and *LIVER INT* (*n* = 286). Besides, there were four journals in the Q1 JCR (Journal Citation Reports) division and the impact factor (IF) of *J HEPATOL* (IF = 25.083) was the highest. Of all the included journals, the top 1,000 journals with the greatest total link strength were selected to make the density map (Figure 4A), which could display the productive journals clearly.

**TABLE 2:** The top 10 journals and co-cited journals associated with the treatment of liver fibrosis.

Journal	Count	IF (2020)	JCR (2020)	Co-cited journal	Citation	IF (2020)	JCR (2020)
HEPATOLOGY	454	17.425	Q1	HEPATOLOGY	71145	17.425	Q1
WORLD J GASTROENTEROL	419	5.742	Q2	J HEPATOL	40572	25.083	Q1
J HEPATOL	393	25.083	Q1	GASTROENTEROLOGY	30634	22.682	Q1
PLOS ONE	381	3.24	Q2	NEW ENGL J MED	14489	91.253	Q1
LIVER INT	286	5.828	Q2	J BIOL CHEM	11136	5.157	Q2
J VIRAL HEPAT	232	3.728	Q2/Q3	GUT	10183	23.059	Q1
HEPATOL RES	189	4.288	Q2	PLOS ONE	9743	3.24	Q2
SCI REP	187	4.38	Q1	J CLIN INVEST	9172	14.808	Q1
J GASTROENTEROL HEPATOL	178	4.029	Q2	LANCET	9125	79.323	Q1
INT J MOL SCI	171	5.924	Q1/Q2	WORLD J GASTROENTEROL	8953	5.742	Q2

Annotations: IF, impact factor; JCR, journal citation reports.

As for the most frequently cited journals in Table 2, the journal *HEPATOLOGY* (*n* = 71145) ranked first, followed by *J HEPATOL* (*n* = 40572), *GASTROENTEROLOGY* (*n* = 30634), *NEW ENGL J MED* (*n* = 14489), and *J BIOL CHEM* (*n* = 11136). In addition, seven journals were located in the Q1 JCR region, and the journal with the highest IF was *NEW ENGL J MED* (IF = 91.253). The density map of Figure 4B well presented the co-cited journals with the top 1,000 selected by the greatest total link strength.

**FIGURE 5:**
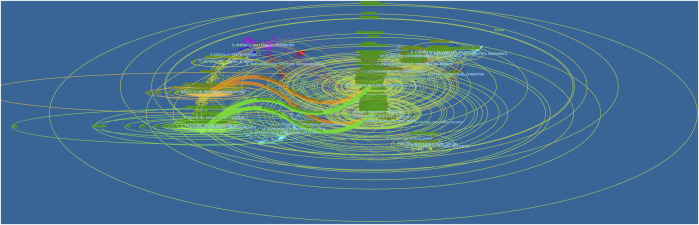
The dual-map overlay of journals associated with the treatment of liver fibrosis (Left: the citing journals; Right: the cited journals).jpg.

The dual-map overlay of journals, characterized by the citing journals on the left and the cited journals on the right, can well show the topic distribution of academic journals (Chen and Leydesdorff, 2014). The relationship between citing and cited journals can be found on the colored paths. As shown in Figure 5, four main reference paths indicating studies published in Molecular/Biology/Genetics and Health/Nursing/Medicine journals were mainly cited by the studies published in Molecular/Biology/Immunology and Medicine/Medical/Clinical journals.

### Productive Authors and Co-Cited Authors

**FIGURE 6:**
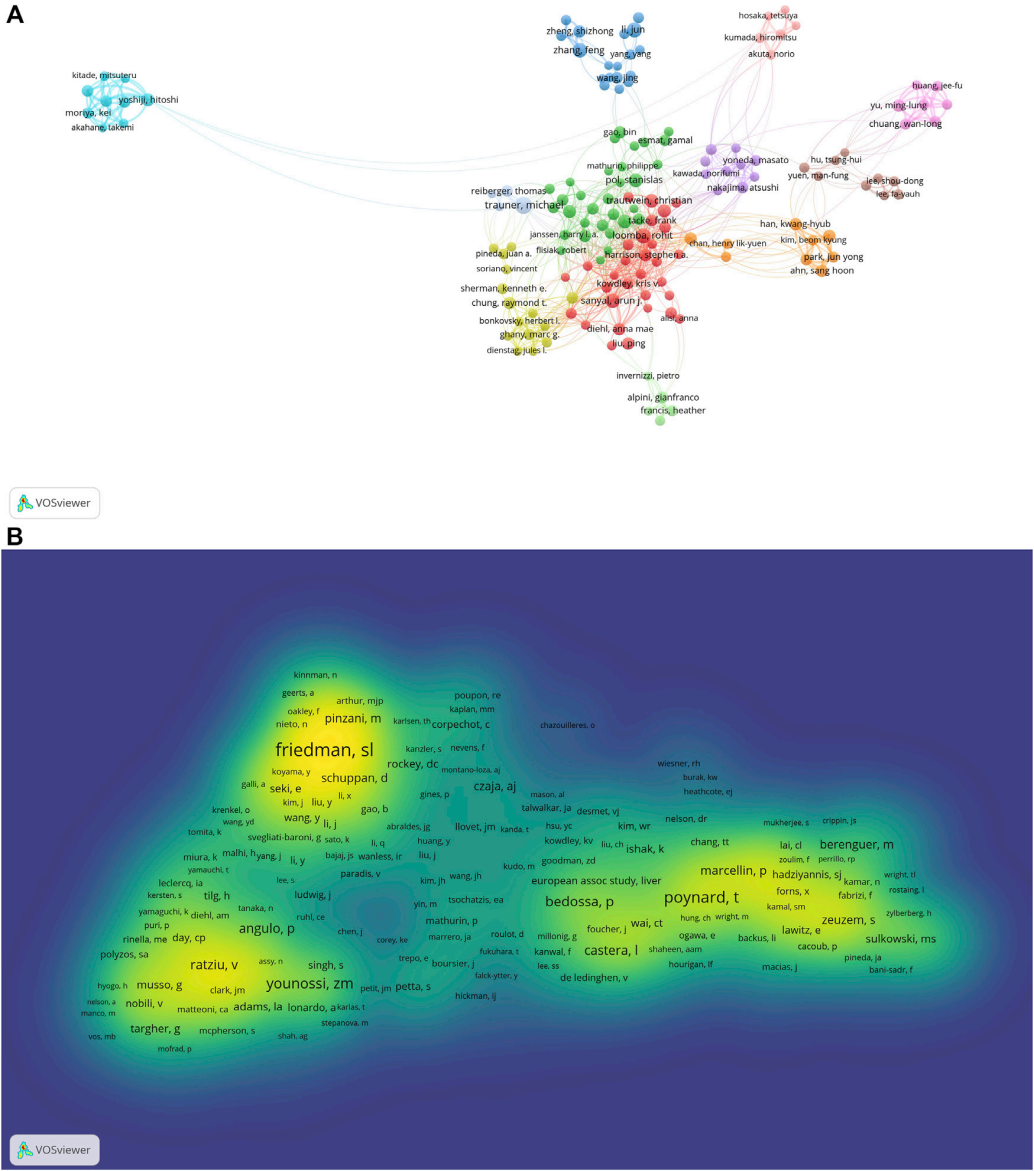
The co-occurrence maps in the treatment of liver fibrosis **(A)** Authors; **(B)** Co-authors. Notes: The size of node indicates the author’s co-occurrence frequencies while its different colors reflect different clusters, and the links reflect the co-occurrence relationship between authors (Map A). The size of word and round, and the opacity of yellow are positively associated with the co-cited frequency (Map B).jpg.

Based on VOSviewer (version 1.6.18), results of the bibliometric analysis showed that there were 72,686 authors retrieved in terms of the treatment of liver fibrosis. As shown in Table 3, Trauner Michael counted top (*n* = 70) in terms of number of published papers, followed by Li Jun (*n* = 56), Loomba Rohit (*n* = 53), Sanyal Arun J (*n* = 48), and Ratziu Vlad (*n* = 48). Meanwhile, according to the choose thresholds that the minimum number of documents of an author was 20, 159 authors were finally selected to draw the network map. As exhibited in Figure 6A, different colors represented different clusters, indicating that there was close cooperation among clusters such as Loomba Rohit and Ratziu Vlad, Loomba Rohit and Nakajima Atsushi, Loomba Rohit and Shiffman Mitchell L, etc. Furthermore, active collaborations were easily found especially among authors in the same cluster, such as Liu Ping and Dieh anna Mae, Trauner Michael and Reiberger Thomas, Zhang Feng and Zheng Shizhong, etc.

**TABLE 3:** The top 10 authors and co-cited authors related to the treatment of liver fibrosis.

Rank	Author	Document	Co-cited author	Citation
1	Trauner Michael	70	Scott L. Friedman	3551
2	Li Jun	56	Poynard Thierry	2424
3	Loomba Rohit	53	Zobair M. Younossi	1801
4	Sanyal Arun J	48	Ramón Bataller	1769
5	Ratziu Vlad	48	Pierre Bedossa	1565
6	Pol Stanislas	45	Lauren, Castéra	1397
7	Trautwein Christian	45	Paul Angulo	1337
8	Schuppan Detlef	44	Vlad Ratziu	1317
9	Zeuzem Stefan	42	Arun J Sanyal	1180
10	Yoshiji Hitoshi	40	Patrick Marcellin	1065

Co-cited authors are two or more authors who are simultaneously co-cited in a range of studies. A total of 180,372 co-authors were searched out. As displayed in Table 3, all of the top 10 authors were co-cited over 1,000 times. The most frequently one was Scott L. Friedman (*n* = 3551), followed by Poynard Thierry (*n* = 2424), Zobair M. Younossi (*n* = 1801), Ramón Bataller (*n* = 1769), and Pierre Bedossa (*n* = 1565). Additionally, the authors (*n* = 5879) with co-citations of at least 20 were made to draw the density map, which could well show the high-frequency co-cited authors according to gradation of yellow. We could see that the yellow region of Scott L. Friedman was the darkest from Figure 6B, suggesting the author was the most co-cited in this field.

### Keyword Co-Occurrence, Clusters, and Evolution

**FIGURE 7:**
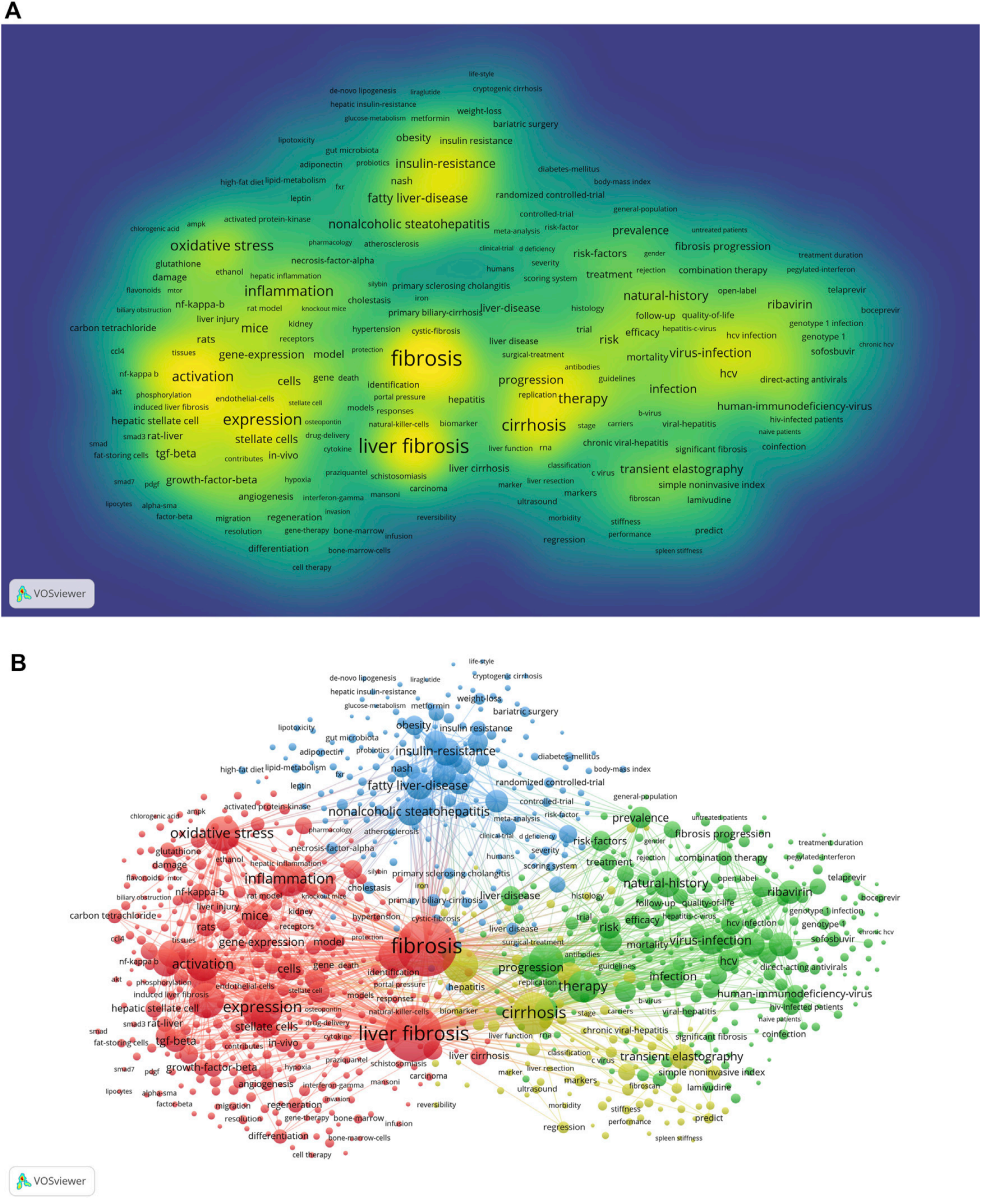
Maps of keywords in the treatment of liver fibrosis **(A)** The density map; **(B)** Co-occurrence network and clusters. Notes: The size of word and round, and the opacity of yellow are positively associated with the co-cited frequency (Map A). The size of the node and keyword indicates the co-occurrence frequencies while their different colors reflect different clusters, and the links reflect the co-occurrence relationship (Map B).jpg.

VOSviewer (version 1.6.18) can provide us with keyword co-occurrence and network cluster analysis. Based on this software, a total of 31,500 keywords were extracted. Considering less influence on a few occurrences of a keyword, we set “minimum number of occurrences of a keyword≧20” as our chosen threshold. Ultimately, 1174 keywords met the threshold and were used for subsequent analysis. The top 20 occurring keywords can be seen in Table 4, which indicated the hotspots of the treatment of liver fibrosis. Meanwhile, 12 keywords appeared more than 1000 times. In terms of frequency, the keyword “fibrosis” ranked first (*n* = 4004), followed by “liver fibrosis” (*n* = 3621), “cirrhosis” (*n* = 2030), “expression” (*n* = 1903), “disease” (*n* = 1382), “hepatic stellate cells” (*n* = 1373), “oxidative stress” (*n* = 1355), “hepatocellular carcinoma” (*n* = 1330), “inflammation” (*n* = 1297), and “activation” (*n* = 1266). Moreover, these high-frequency keywords were intuitively shown in the density map (Figure 7A).

**TABLE 4:** The top 20 keywords associated with the treatment of liver fibrosis.

Rank	Keyword	Count	Rank	Keyword	Count
1	Fibrosis	4004	11	Therapy	1159
2	Liver fibrosis	3621	12	Liver	1146
3	Cirrhosis	2030	13	Apoptosis	875
4	Expression	1903	14	Insulin-resistance	873
5	Disease	1382	15	Injury	839
6	Hepatic stellate cells	1373	16	Nonalcoholic steatohepatitis	825
7	Oxidative stress	1355	17	Virus infection	818
8	Hepatocellular-carcinoma	1330	18	Fatty liver disease	813
9	Inflammation	1297	19	Mechanisms	787
10	Activation	1266	20	Natural history	771

In addition, Figure 7B presented a result of network cluster analysis on keywords. In this map, there were 4 clusters representing 4 research directions and scopes. Among them, cluster 1 painted by red was the largest one, followed by cluster 2 (green), cluster 3 (blue), and cluster 4 (yellow). Specifically, in cluster 1, there were 429 items including liver fibrosis, inflammation, oxidative stress, hepatic stellate cell, activation, etc. In cluster 2, there were 270 items including therapy, direct-acting antivirals, ribavirin, sofosbuvir, fibrosis progression, etc. In cluster 3, there were 196 items including nonalcoholic steatohepatitis (NASH), fatty liver disease, insulin-resistance, obesity, body-mass index, etc. In cluster 4, there were 105 items including cirrhosis, transient elastography, chronic viral hepatitis, liver function, fibroscan, etc.

**FIGURE 8:**
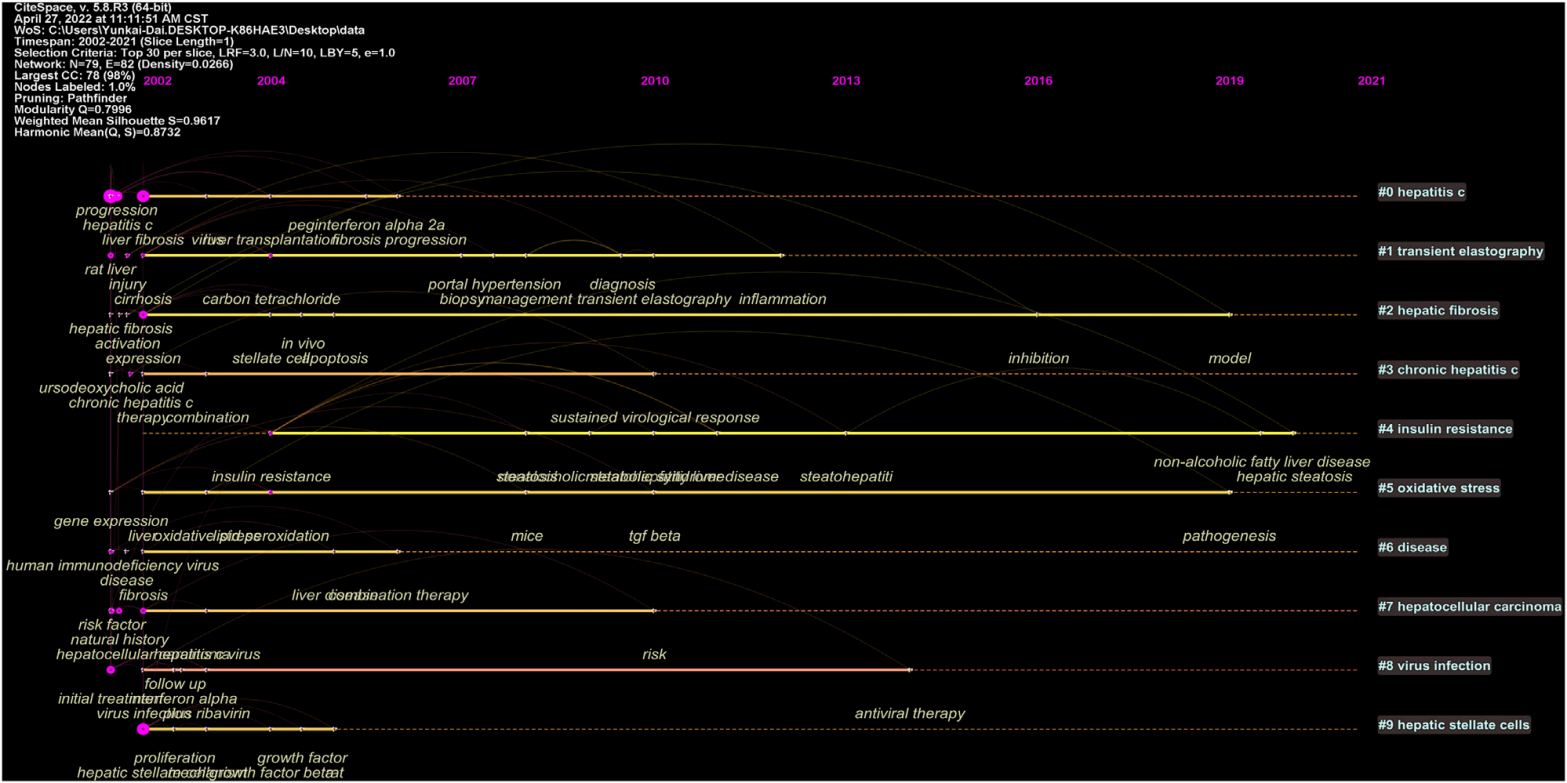
Keywords timeline viewer of the treatment of liver fibrosis.jpg.

Keywords timeline viewer was built by CiteSpace, which could cluster keywords and take time into consideration, contributing to well showing the evolution of high-frequency keywords in each cluster. Furthermore, the viewer could help us easily find the period of a particular topic and the evolution track of our research field. As displayed in Figure 8, the focus of the treatment of liver fibrosis at each stage and evolution track were intuitively seen.

### Co-Cited References and Reference Burst

In order to detect the most co-cited references and reference bursts related to the treatment of liver fibrosis, CiteSpace [version 5.8.R3 (64-bit)] was used for bibliometric analysis. As shown in Table 5, all the included references were clustered as three relevant parts (A. chronic hepatitis b, B. liver fibrosis, and C. antifibrotic effects) and the top 10 co-cited references were respectively associated with the three parts (Friedman, 2003; Bataller and Brenner, 2005; Gressner and Weiskirchen, 2006; Iredale, 2007; Seki et al., 2007; Friedman, 2008a; Friedman, 2008b; Schuppan and Afdhal, 2008; Wynn, 2008; European Association For The Study Of The Liver, 2009; Lok and Mcmahon, 2009; Friedman, 2010; European Association For The Study Of The Liver, 2012; Marcellin et al., 2013; Schuppan and Kim, 2013; Afdhal et al., 2014a; Afdhal et al., 2014b; Poordad et al., 2014; EASL-ALEH Clinical Practice Guidelinesm, 2015; Lee et al., 2015; Seki and Schwabe, 2015; Trautwein et al., 2015; Sarin et al., 2016; Terrault et al., 2016; Bachofner et al., 2017; Higashi et al., 2017; Koyama and Brenner, 2017; Tsuchida and Friedman, 2017; Terrault et al., 2018). Of the three parts, an article published by Tsuchida et al., in 2017 ranked first with the most co-cited references (260 citations) and elaborated mechanisms of hepatic stellate cell (HSC) activation (Chen, 2017). Meanwhile, part A, clustered as chronic hepatitis b, had 9 co-cited references with the description of nucleoside/nucleotide analogs being representative of antiviral agents (European Association For The Study Of The Liver, 2009; Lok and Mcmahon, 2009; European Association For The Study Of The Liver, 2012; Marcellin et al., 2013; Sarin et al., 2016; Terrault et al., 2016; Bachofner et al., 2017; Terrault et al., 2018). Although parts B and C were respectively clustered as liver fibrosis and antifibrotic effect, a nominal term “HSC” was co-occurred between them. The only difference was that the term was focused on the pathogenesis of liver fibrosis in part B (Schuppan and Kim, 2013; Lee et al., 2015; Seki and Schwabe, 2015; Trautwein et al., 2015; Higashi et al., 2017; Koyama and Brenner, 2017; Tsuchida and Friedman, 2017) while part C focused on finding the antifibrotic effects (Friedman, 2003; Bataller and Brenner, 2005; Gressner and Weiskirchen, 2006; Iredale, 2007; Seki et al., 2007; Friedman, 2008a; Friedman, 2008b; Wynn, 2008; Friedman, 2010). Besides, among the three top 10 co-cited references, the most frequent journal was *J HEPATOL*, followed by *HEPATOLOGY* and *J CLIN INVEST*.

**TABLE 5:** The top 10 co-cited references associated with the treatment of liver fibrosis.

A. Top 10 co-cited references associated with chronic hepatitis b						
Rank	Citation	Title	First author	Year	Journal	Centrality
1	169	EASL 2017 Clinical Practice Guidelines on the management of hepatitis B virus infection	European Assoc Study Liver	2017	J HEPATOL	0.01
2	128	EASL Clinical Practice Guidelines: Management of chronic hepatitis B virus infection	European Assoc Study Liver	2012	J HEPATOL	0.04
3	114	Regression of cirrhosis during treatment with tenofovir disoproxil fumarate for chronic hepatitis B: a 5-year open-label follow-up study	Marcellin P	2013	LANCET	0.02
4	109	EASL-ALEH Clinical Practice Guidelines: Non-invasive tests for evaluation of liver disease severity and prognosis	European Assoc Study Liver	2015	J HEPATOL	0.01
5	96	Asian-Pacific clinical practice guidelines on the management of hepatitis B: a 2015 update	Sarin SK	2016	HEPATOL INT	0.02
6	80	Update on prevention, diagnosis, and treatment of chronic hepatitis B: AASLD 2018 hepatitis B guidance	Terrault NA	2018	HEPATOLOGY	0
7	76	Chronic hepatitis B: Update 2009†	Lok ASF	2009	HEPATOLOGY	0.18
8	63	AASLD guidelines for treatment of chronic hepatitis B	Terrault NA	2016	HEPATOLOGY	0.03
9	63	Direct antiviral agent treatment of chronic hepatitis C results in rapid regression of transient elastography and fibrosis markers fibrosis-4 score and aspartate aminotransferase-platelet ratio index	Bachofner JA	2017	LIVER INT	0
10	60	EASL Clinical Practice Guidelines: Management of chronic hepatitis B	European Assoc Study Liver	2009	J HEPATOL	0.06
B. Top 10 co-cited references associated with liver fibrosis						
1	260	Mechanisms of hepatic stellate cell activation	Tsuchida T	2017	NAT REV GASTRO HEPAT	0.04
2	145	Hepatic stellate cells as key target in liver fibrosis	Higashi T	2017	ADV DRUG DELIVER REV	0.01
3	128	Pathobiology of liver fibrosis: a translational success story	Lee YA	2015	GUT	0.06
4	107	Ledipasvir and sofosbuvir for untreated HCV genotype 1 infection	Afdhal N	2014	NEW ENGL J MED	0.42
5	102	Ledipasvir and Sofosbuvir for Previously Treated HCV Genotype 1 Infection	Afdhal N	2014	NEW ENGL J MED	0.18
6	101	Liver inflammation and fibrosis	Koyama Y	2017	J CLIN INVEST	0
7	97	Hepatic inflammation and fibrosis: Functional links and key pathways	Seki E	2015	HEPATOLOGY	0
8	93	Evolving therapies for liver fibrosis	Schuppan D	2013	J CLIN INVEST	0.06
9	86	Hepatic fibrosis: Concept to treatment	Trautwein C	2015	J HEPATOL	0.05
10	74	ABT-450/r–Ombitasvir and Dasabuvir with Ribavirin for Hepatitis C with Cirrhosis	Poordad F	2014	NEW ENGL J MED	0.03
C. Top 10 co-cited references associated with antifibrotic effects						
1	205	Liver fibrosis	Bataller R	2005	J CLIN INVEST	0.16
2	203	Mechanisms of Hepatic Fibrogenesis	Friedman SL	2008	GASTROENTEROLOGY	0.06
3	99	Hepatic Stellate Cells: Protean, Multifunctional, and Enigmatic Cells of the Liver	Friedman SL	2008	PHYSIOL REV	0.02
4	87	Liver fibrosis—from bench to bedside	Friedman SL	2003	J HEPATOL	0
5	59	Modern pathogenetic concepts of liver fibrosis suggest stellate cells and TGF-β as major players and therapeutic targets	Gressner AM	2006	J CELL MOL MED	0.01
6	51	Models of liver fibrosis: exploring the dynamic nature of inflammation and repair in a solid organ	Iredale JP	2007	J CLIN INVEST	0.07
7	42	Cellular and molecular mechanisms of fibrosis†	Wynn TA	2008	J PATHOL	0.01
8	39	Evolving challenges in hepatic fibrosis	Friedman SL	2010	NAT REV GASTRO HEPAT	0.01
9	29	TLR4 enhances TGF-β signaling and hepatic fibrosis	Seki E	2007	NAT MED	0
10	24	Liver cirrhosis	Schuppan D	2008	LANCET	0.01

**FIGURE 9:**
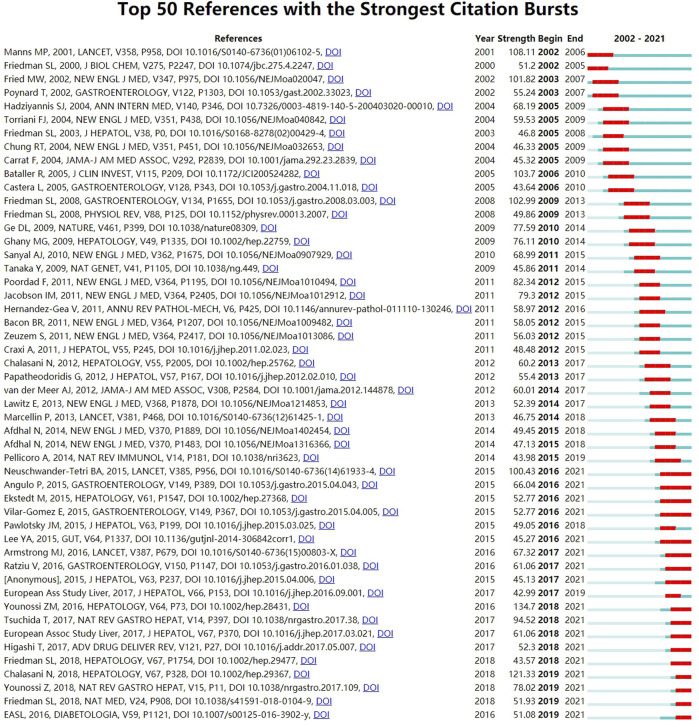
View Citation Burst History. Notes: The blue bars mean the reference had been published; The red bars mean citation burstness.jpg.

Reference burst, characterized by being cited frequently over a while (Chen 2017), was detected by CiteSpace software with the selection criteria which included 2 years at least on the burst duration and references with the strongest citation bursts. As displayed in Figure 9, there were 6 references (12%) appeared in citation burstness in 2016 and 2012, followed by 2018 and 2005 (both had five references accounting for 10%). Additionally, it was noted that 17 references accounting for 34% were in burstness until 2021. As for the strongest burstness, a review entitled “Global epidemiology of nonalcoholic fatty liver disease-Meta-analytic assessment of prevalence, incidence, and outcomes” with the strength of 134.7 was published in *HEPATOLOGY* by Younossi et al. (2016), etc. in 2016 (Younossi et al., 2016). Moreover, its citation burstness was from 2018 to 2021.

## Discussion

Liver fibrosis is shown in most chronic liver diseases caused by hepatitis virus, alcohol, drugs, schistosome, autoimmune hepatitis (AIH), primary biliary cholangitis (PBC), primary sclerosing cholangitis (PSC), NASH, AFLD, and NAFLD, etc (Higashi et al., 2017). It is the unavoidable pathological process for the development of chronic liver disease to liver cirrhosis. Liver fibrosis is also the damage and inflammation in the liver caused by various pathogenic factors, resulting in extensive hyperplasia and deposition of fibrotic tissue, which is the common pathological basis of various chronic liver diseases. Hepatocyte cell death can trigger capillarization of liver sinusoidal endothelial cells, stimulation of immune cells (including macrophages and Kupffer cells), and activation of HSCs, leading to the progression of liver fibrosis (Odagiri et al., 2021). Furthermore, activated HSCs are the key effectors of fibrogenesis through increased deposition of fibrillar ECM and by releasing cytokines, chemokines, and other mediators, together with inflammatory cells, establishing a pro-fibrogenic environment that negatively affects the regeneration of the liver parenchyma (Friedman and Pinzani, 2022). Cytokines, such as transforming growth factor-β (TGF-β), platelet-derived growth factor (PDGF), connective tissue growth factor (CTGF), and vascular endothelial growth factor (VEGF), can regulate the cell signal transduction mechanism of autocrine paracrine activation of HSCs, and their HSCs function in ECM degradation and metabolism through the expression of tissue inhibitors of matrix metalloproteinases (TIMPs). Although that TGF-β can stimulate the activation of HSCs are common mechanisms, schistosomiasis fibrosis is mainly caused by IL-13/Stat while NASH is mostly caused by lipid toxicity metabolism and lipid peroxidation-related stimulation.

The development of modern hepatology technology has changed the natural history of many chronic liver diseases, including not only liver fibrosis but also a certain degree of cirrhosis. In certain animal experiments, removing the causative agents resulted in cirrhosis regression (Ramachandran and Iredale, 2009). Effective fibrolysis requires the targeting of several mechanisms, including 1) ECM degradation, 2) myofibroblast deactivation, 3) hepatocyte regeneration, and 4) vascular and parenchymal remodeling (Jangra et al., 2022). For example, liver injury initiates the transdifferentiation of quiescent HSCs to their activated phenotype characterized by specific phenotypic changes including proliferation, contractility, fibrogenesis, altered matrix degradation, chemotaxis, and inflammatory signaling. During the resolution of hepatic fibrosis, activated HSCs can be cleared by apoptosis or reversion to an inactivated phenotype (Tsuchida and Friedman, 2017). As for different etiological and symptomatic treatments, the corresponding drug targets were concluded as follows (Friedman, 2015; Yoon et al., 2016; Odagiri et al., 2021; Jangra et al., 2022): 1) NASH: pan-caspase inhibitors, apoptosis signal-regulating kinase 1 (ASK1) inhibitors, pirfenidone, fibroblast growth factor 21 (FGF21), lipid-lowering agents, stearoyl-coenzyme A desaturase 1 (SCD1) inhibitors, thyroid hormone receptor-β (THR-β) agonists, and FGF 19 analogs; AIH: corticosteroids, C-C chemokine receptors 2 and 5 (CCR2/CCR5) inhibitors, galectin-3 inhibitors; PBC: urso deoxycholic acid (UDCA). 2) HBV and HCV: tenofovir, entecavir, interferon, direct-acting antivirals (DAAs); Reduction of fibrotic scar evolution: lysyl oxidase-like 2 (LOXL2) inhibitors and heat shock protein 47 (HSP47) siRNA. 3) peroxisome proliferator-activated receptor-γ (PPARγ) agonists, farnesoid X receptor (FXR) agonists, CREB-binding protein (CBP)/β-catenin inhibitors, NADPH oxidase (NOX) 1/4 inhibitors, and nitazoxanide. 4) ECM inhibitors.

However, effective biological or chemical drugs for fibrosis treatment are not yet available for clinical use. The development of effective therapies requires to understand further mechanisms underlying the fibrogenic process.

### General Information

According to our selection criteria from 2002 to 2021 on WoSCC database, a total of 15,237 studies associated with the treatment of liver fibrosis were published in different academic journals (*n* = 2017) by 72,686 authors in 200 institutions from 134 countries/regions.

Through a bibliometric and visual analysis of the relevant research literature, we found a continuously increasing number of publications from 2002 to 2021 (Figure 2), indicating that the annual output grew steadily and this field research had gained researchers’ interest before 2002. For the 20 years, the number of literature had increased year by year and a large number of researchers committed to the treatment of liver fibrosis, subsequently creating more valuable scientific achievements, meaning that there may be some key issues to be solved in this field. Therefore, it remains a potential area of research and is worth investing a lot of material resources and manpower, which is consistent with the actual clinical situation.

Analysis of countries/regions and institutions distribution helps to facilitate teamwork and global collaboration in a certain field. In this study, we could see the distribution that the country with the most publications was United States (*n* = 3687), followed by China (*n* = 3254), and Japan (*n* = 1057) (Table 1). Moreover, United States had the highest centrality with 0.84, indicating that it played an important role as a bridge in the cooperation between countries. In addition, the institution with the most publications was Shanghai Jiao Tong University (*n* = 201), followed by the University of California San Diego (*n* = 191), and Mayo Clinic (*n* = 190) (Table 1), indicating researchers from the United States and China were core research forces and the two countries were the most influential in terms of the treatment of liver fibrosis (Patel et al., 2006; Yin et al., 2007; Veldt et al., 2008; Jin et al., 2015). This distribution may be associated with the economic development and financial put into academic research of these countries. Besides, four-fifths of the top 10 institutions were from China and United States, suggesting their notable contributions to this field. Meanwhile, the co-occurrence map of countries from Figure 3A showed there were lots of extensive and close collaborations especially United States and China. The co-occurrence map of institutions from Figure 3B indicated the year 2015 and after were the most intensive inter-institution cooperation, but the before had rare inter-institution cooperation. Considering the above, it was important to strengthen the exchanges and cooperation between countries and institutions to promote further development of this field and benefit more liver fibrosis sufferers.

Journals and co-cited journals analysis can help researchers select the appropriate journals for paper submission. As shown in Table 2 and Figures 4A,B, *HEPATOLOGY* was both the most published journal (*n* = 454) and the most frequently co-cited journal (*n* = 71145). Of the top 10 journals, there were 4 in the Q1 JCR division, and the journal with the highest IF was *J HEPATOL* (IF = 25.083). Of the top 10 co-cited journals, there were 7 located in the Q1 JCR division, and the highest IF was *NEW ENGL J MED* (IF = 91.253). These distributions suggested that research on the treatment of liver fibrosis was favored by many high-quality and high-impact journals. Furthermore, results of the dual-map overlay of journals showed that documents published in Molecular/Biology/Genetics and Health/Nursing/Medicine journals were mainly cited in those published in Molecular/Biology/Immunology and Medicine/Medical/Clinical journals (Figure 5), which indicated the study on this field primarily focused on basic research and translational medicine.

Highlighting the authors with numerous co-occurrences or co-cited publications in a certain field can contribute to moving along the road and providing further guidelines (Kodonas et al., 2021). In our analysis, we can obtain information about potential collaborators and influential research groups from Figure 6. Table 3 showed that Trauner Michael was the most frequently published author (*n* = 70) and Scott L. Friedman had the most co-citations (*n* = 3551), indicating they had a potential outstanding contribution to this field.

### Knowledge Base

Co-cited references, cited together by other publications, can measure the degree of relevance among studies. And the knowledge base, characterized by the corresponding research community, is a collection of co-cited references (Chen 2017). Moreover, research papers with the highest co-citation frequency are important research bases in a certain field. As shown in Table 5, the top co-cited 10 references, clustered by three parts (A. chronic hepatitis b, B. liver fibrosis, and C. antifibrotic effects), were included because of being more related to the treatment of liver fibrosis and as follows:

In terms of viral hepatitis (part A), four titles respectively named “EASL 2017 Clinical Practice Guidelines on the management of hepatitis B virus infection” (2017), “EASL Clinical Practice Guidelines: Management of chronic hepatitis B virus infection” (2012), “EASL Clinical Practice Guidelines: Management of chronic hepatitis B” (2009), and “EASL-ALEH Clinical Practice Guidelines: Non-invasive tests for evaluation of liver disease severity and prognosis” (2015) were all written by European Assoc Study Liver and published all in *J HEPATOL* (European Association For The Study Of The Liver, 2009; European Association For The Study Of The Liver, 2012; EASL-ALEH Clinical Practice Guidelines. 2015; European Association For The Study Of The Liver, 2017). The first three guidelines presented updated recommendations for the optimal management of hepatitis B virus (HBV) infection on before. In order to improve survival and quality of life, long-term suppression of HBV replication, including nucleoside/nucleotide analogs such as entecavir, tenofovir disoproxil, or tenofovir alafenamide, was the main goal of current treatment strategies. And the last guideline summarized two currently available non-invasive approaches: one was a “biological” method with the aid of quantification of biomarkers in serum specimens and the other was a “physical” method by means of the measurement of liver stiffness. Another clinical practice guideline involving in Asian-Pacific region was published in *HEPATOL INT* by Sarin et al. (2016). In terms of the treatment of liver fibrosis, this guideline summarized that preventing the progression of the disease to cirrhosis or even HCC started from the eradication of HBV infection, which could also improve survival and quality of life. Additionally, another two guidelines from the American Association for the Study of Liver Diseases (AASLD) published in *HEPATOLOGY* were all written by Terrault et al. (Terrault et al., 2016; Terrault et al., 2018). The guideline in 2018 (Terrault et al., 2018) complemented that in 2016 and updated the previous HBV and CHB guidelines in 2009 (Lok and Mcmahon, 2009), briefly including prevention, diagnosis, and treatment. Moreover, an article published in a 5-year open-label follow-up study in *LANCET* in 2013 was about to assess the efficacy of tenofovir disoproxil fumarate (DF) for fibrosis and cirrhosis in chronic HBV infection (Marcellin et al., 2013). And it was proved that long-term suppression of HBV, such as taken administration of tenofovir DF, could be contributed to regression of fibrosis and cirrhosis. After 3 years to 2016, Bachofner et al. (2017) published an article titled “Direct antiviral agent treatment of chronic hepatitis C results in rapid regression of transient elastography and fibrosis markers fibrosis-4 score and aspartate aminotransferase-platelet ratio index” in *LIVER INT*, which concluded that sufferers with a sustained virological response after direct antiviral agents therapy showed that significant regression of transient elastography (TE) values and rapid decrease in TE were consistent with regression of validated fibrosis-4 and aspartate aminotransferase-platelet ratio index scores (Bachofner et al., 2017).

Although parts B and C were respectively clustered as liver fibrosis and antifibrotic effect, a nominal term “HSC” was co-occurred between them. The only difference was that the term was focused on the pathogenesis of liver fibrosis in part B (Schuppan and Kim, 2013; Lee et al., 2015; Seki and Schwabe, 2015; Trautwein et al., 2015; Higashi et al., 2017; Koyama and Brenner, 2017; Tsuchida and Friedman, 2017) while part C focused on finding the antifibrotic effects (Friedman, 2003; Bataller and Brenner, 2005; Gressner and Weiskirchen, 2006; Iredale, 2007; Seki et al., 2007; Friedman, 2008a; Friedman, 2008b; Wynn, 2008; Friedman, 2010). Besides, in part B, Afdhal et al. published two articles involving in the clinical efficacy of ledipasvir and sofosbuvir from *NEW ENGL J MED* (Afdhal et al., 2014a; Afdhal et al., 2014b). One was aimed at untreated HCV genotype 1 infection while the other was at previously treated HCV genotype 1 infection. But both of them showed good clinical efficacy among patients. Poordad et al. (2014) published clinical research titled “ABT-450/r–Ombitasvir and Dasabuvir with Ribavirin for Hepatitis C with Cirrhosis” in *NEW ENGL J MED* (Poordad et al., 2014). The research evaluated the efficacy of these drugs combination for previously untreated and treated adults with HCV genotype 1 infection and compensated cirrhosis. In part C, a publication titled “Liver cirrhosis” was written by Schuppan D and published in *LANCET* (Schuppan and Afdhal, 2008), which summarized the diagnosis, complications, treatment of cirrhosis, and new clinical and scientific developments.

### The Analysis of Hotspot Evolution, Knowledge Structure, and Emerging Topics

Keywords co-occurrence, in bibliometrics, can reflect the hotspots and directions of an academic field (Xiao et al., 2017). As shown in Table 4, the top 20 keywords with the high-frequency terms involving in the treatment of liver fibrosis contained liver fibrosis, cirrhosis, HSC, oxidative stress, HCC, inflammation, activation, therapy, apoptosis, insulin-resistance, NASH, virus infection, fatty liver disease, mechanisms, and natural history. From these representative terms, we can generalize the general situation of this research field. Specifically, 1) the natural history of liver fibrosis can develop into cirrhosis or even HCC; 2) the pathogenesis of liver fibrosis is associated with virus infection, inflammation, oxidative stress, activation of HSC, hepatocellular apoptosis, which are both therapeutic targets and pathological mechanisms in terms of liver fibrosis (Lee et al., 2015; Seki and Schwabe, 2015; Higashi et al., 2017; Koyama and Brenner, 2017; Tsuchida and Friedman, 2017); 3) some certain primary etiologies and chronic underlying diseases such as insulin-resistance, NASH, and fatty liver disease should not be ignored; 4) long-term suppression of virus replication, namely taking administration of nucleoside/nucleotide analogs such as entecavir, tenofovir disoproxil or tenofovir alafenamide, is the main goal of current treatment strategies (2017; 2012; 2015).

Both densities of map and network clustering analysis of keywords can respectively intuitively show the high-frequency keywords and research directions in the treatment of liver fibrosis (Figures 7A,B). There were 4 clusters colored by red, green, blue, and yellow in Figure 7B. Cluster 1 was primarily concerned with the pathogenesis of liver fibrosis, as well as the therapeutic targets. Cluster 2 was involved with the treatment strategies and evaluation of fibrosis progression. Cluster 3 was mainly about primary etiologies and chronic basic diseases. Cluster 4 mainly covered biomarkers and indexes of liver fibrosis and liver function regression. Timeline viewers about keywords, as shown in Figure 8, can display the evolution of new hotspots in this field (Liu et al., 2014). According to this map, we can see the time of a topic and explore the evolutionary trajectory in the treatment of liver fibrosis.

Emerging research topics of a certain field could be also characterized by references with intense citation bursts (Chen 2017). In this bibliometrics, there were 271 references having citation bursts and the top 50 were selected in Figure 9. A meta-analysis, with the most powerful citation bursts (Strength: 134.7), was conducted by Younossi et al. (2016) and published in *HEPATOLOGY* (Younossi et al., 2016), which evaluated the prevalence, incidence, and outcomes of NAFLD, showing that fibrosis progression proportion and mean annual rate of progression in NASH were 40.76% and 9%. More importantly, the findings indicated NASH patients being the onset of cirrhosis would increase their odds of developing HCC. In addition to this meta-analysis, there were another 16 references including 5 (2017; 2015; Higashi et al., 2017; Tsuchida and Friedman, 2017; Lee et al., 2015) discussed above in the knowledge base of Table 5, which were all still in a state of citation burst and represented the latest emerging topics. Then the remaining 11 references, based on a ranking by burstness strength (from high to low), were as follows: the first paper (strength = 121.33) was published by AASLD (Chalasani et al., 2018) in *HEPATOLOGY*. This practice guidance was an update to the diagnostic, therapeutic, and preventive aspects of NAFLD care, which also proved that NASH patients with fibrosis had higher risk of cirrhosis and liver-related mortality. The second paper (strength = 100.43) was published by Neuschwander-Tetri et al. (2015) in *LANCET*, which summarized the efficacy of obeticholic acid in sufferers with NASH and showed that the acid could improve its histological features. The third paper (strength = 78.02) was a review published in Younossi et al. (2018) in *NAT REV GASTRO HEPAT*. It generalized the trends, predictions, risk factors, and prevention of NAFLD and NASH, which suggested that the progression of NAFLD from steatosis to NASH and fibrosis was not linear, and the prognostic factor of liver-related diseases and mortality were closely associated with fibrosis. The fourth paper (strength = 67.32) was an article published by Armstrong et al. (2016) in *LANCET*, which concluded that the efficacy and safety of liraglutide for the treatment of NASH were positive with a multicenter, double-blind, randomized, placebo-controlled phase 2 study. The fifth paper (strength = 66.04) was a longitudinal study conducted by Angulo et al. (2015) and published in *GASTROENTEROLOGY*. Results showed that the fibrosis stage without other histologic features of steatohepatitis was independently related to long-term liver transplantation and liver-related events. The sixth paper (strength = 61.06) was published by Ratziu et al. (2016) in *GASTROENTEROLOGY*. This study introduced that elafibranor could induce resolution of NASH without fibrosis worsening. The seventh paper (strength = 52.77) was published by Vilar-Gomez et al. (2015) in *GASTROENTEROLOGY*. This study proved that losing weight induced by lifestyle changes could significantly improve the histologic features of NASH. The eighth paper (strength = 52.77) was published by Ekstedt et al. (2015) in *HEPATOLOGY*. After as many as 33 years of follow-up, this article mainly proved that fibrosis stage was the strongest predictor for disease-specific mortality in NAFLD. The ninth paper (strength = 51.93) was a review written by Friedman et al. (2018a) in *NAT MED*. This publication mainly introduced the pathogenic and clinical features of NAFLD, and could enable refinement of therapeutic targets based on animal models of NAFLD. Therefore, it could accelerate drug development. The 10th paper (strength = 51.08) was published by the European Association for the Study of the Liver (EASL), European Association for the Study of Diabetes (EASD), and European Association for the Study of Liver (2016) in *DIABETOLOGIA*. The guideline mainly proposed recommendations for the management of NAFLD including diagnosis, treatment, and follow-up. For the treatment, diet and lifestyle changes for all patients should be recommended. And progressive sufferers including bridging fibrosis and cirrhosis but also for early-stage NASH with high risk of fibrosis progression or active-stage with increased necroinflammatory activity should be recommended for drug treatments. The 11th paper (strength = 43.57) was a randomized, placebo-controlled trial conducted by Friedman et al. (2018b) in *HEPATOLOGY*. This study mainly explored that cenicriviroc could achieve liver fibrosis improvement and no worsening of steatohepatitis in NASH patients.

From the abovementioned analysis, some important information can be summarized as follows: 1) HBV is still the main cause of liver fibrosis (Inoue and Tanaka, 2020) and long-term suppression of HBV replication is the main goal of current treatment strategies. 2) The activation of HSC is a crucial pathogenesis of liver fibrosis and is also a hotspot in this research field. 3) The progressive liver fibrosis is closely associated with preventive, therapeutic, prognostic factors of liver-related diseases such as NAFLD and NASH. 4) Diet and lifestyle modification apply to patients with NAFLD or NASH. Drug treatments, such as cenicriviroc, liraglutide, elafibranor, etc., can achieve regression of NASH with no fibrosis worsening and also improve liver fibrosis. These conclusions mentioned above are in accordance with the evolution of new hotspots about keywords in timeline viewer (Figure 8).

### Limitations

There are certain limitations inherent in our bibliometrics. First, this bibliometric study was conducted by CiteSpace and VOSviewer, which only analyzed the main conclusions rather than full texts, leading to failing to completely replace the system search. Second, our retrieved publications and data were all from WoSCC. Therefore, some documents excluded from this database should be taken into consideration, and citation counts were probably underestimated. Nevertheless, data from WoSCC could be representative of numerous information to a certain extent in view of the database regarded as the most commonly used one in bibliometric analysis. Finally, all included data and publications were analyzed based on the machine learning and natural language processing, which might result in bias as reported in other bibliometric analyses. Despite these, in this study, all maps based on the retrieved data can intuitively present hotspots, evolution process, and development trends of the treatment of liver fibrosis, which can provide many important reference values for the newcomers to the field. However, if a more specific topic is focused on in the future to sort out more meaningful conclusions for subsequent studies, it will offer more crucial clues and ideas for scholars focusing on this field. This is also the direction of our follow-up efforts.

## Conclusion

In a word, the treatment of liver fibrosis is still a popular topic and keeps a rapid development stage with active collaboration around the world. The United States and China are the dominating cooperating centers. There are summaries of knowledge base and research hotspots as follows:

1) The period of frequent inter-institution cooperation between countries was the years 2015 and after, but rare inter-institution cooperation was noticed on or before. Shanghai Jiao Tong University and United States were the institutions and the country respectively that contributed the most publications.
2) The journal *HEPATOLOGY* was not only the most published one but also the most frequently co-cited journal in terms of the treatment of liver fibrosis.
3) The research hotspots related to liver fibrosis included virus infection, inflammation, oxidative stress, activation of HSC, and hepatocellular apoptosis, which could be also targets of the treatment.
4) The latest hotspots and topics involving in this field focused on the long-term suppression of HBV replication and the activation of HSC. And emerging topics mainly included the studies that treatment of NAFLD and NASH especially NASH can achieve regression of them with no fibrosis worsening and improve liver fibrosis, which was also rapidly developing hot fields.

## Data Availability

The original contributions presented in the study are included in the article/Supplementary Material; further inquiries can be directed to the corresponding authors.
